# Effect of NaCl on Heat Resistance, Antibiotic Susceptibility, and Caco-2 Cell Invasion of *Salmonella*

**DOI:** 10.1155/2013/274096

**Published:** 2013-07-08

**Authors:** Hyunjoo Yoon, Beom-Young Park, Mi-Hwa Oh, Kyoung-Hee Choi, Yohan Yoon

**Affiliations:** ^1^Department of Food and Nutrition, Sookmyung Women's University, Seoul 140-742, Republic of Korea; ^2^National Institute of Animal Science, RDA, Suwon, Gyeonggi 441-706, Republic of Korea; ^3^Department of Oral Microbiology, College of Dentistry, Wonkwang University, Iksan, Chonbuk 570-749, Republic of Korea

## Abstract

This study evaluated the effects of NaCl on heat resistance, antibiotic susceptibility, and Caco-2 cell invasion of *Salmonella*. *Salmonella typhimurium* NCCP10812 and *Salmonella enteritidis* NCCP12243 were exposed to 0, 2, and 4% NaCl and to sequential increase of NaCl concentrations from 0 to 4% NaCl for 24 h at 35°C. The strains were then investigated for heat resistance (60°C), antibiotic susceptibility to eight antibiotics, and Caco-2 cell invasion efficiency. *S. typhimurium* NCCP10812 showed increased thermal resistance (*P* < 0.05) after exposure to single NaCl concentrations. A sequential increase of NaCl concentration decreased (*P* < 0.05) the antibiotic sensitivities of *S. typhimurium* NCCP10812 to chloramphenicol, gentamicin, and oxytetracycline. NaCl exposure also increased (*P* < 0.05) Caco-2 cell invasion efficiency of *S. enteritidis* NCCP12243. These results indicate that NaCl in food may cause increased thermal resistance, cell invasion efficiency, and antibiotic resistance of *Salmonella*.

## 1. Introduction

*Salmonella* spp. are pathogenic bacteria that are responsible for foodborne diseases, with *enteritidis*, *typhimurium*, and Heidelbergare being the serotypes most frequently involved in *Salmonella* foodborne outbreaks [1]. The pathogen possesses the ability to enter and penetrate the human epithelial cell, causing fever, gastroenteritis, and bacteremia [2, 3]. Since antibiotic resistant *Salmonella* has been isolated from various foods, the bacteria have become a worldwide food safety issue [4, 5].

*Salmonella* spp. require high availability of water (*a*_*w*_) ranging between 0.94 and 0.99; however, since the bacteria can still survive below that *a*_*w*_ range, it is a widespread pathogen that has been also isolated from high-salted fish and meat products [3, 6]. A study by Oscar [7] showed that previous exposure of *Salmonella* to NaCl affected the kinetic parameters of the bacteria, such as lag phase duration and maximum specific growth rate. For *Staphylococcus aureus*, exposure to NaCl increased its thermal resistance in ready-to-heat sauces [8]. Other studies also showed that the exposure of bacteria to sublethal stresses such as acid, heat, and NaCl increased their cross-protection responses, especially following continuous exposure to the stresses [9, 10]. A study by Kieboom et al. [11] revealed that *S*. *enteritidis* cells exposed to *a*_*w*_ = 0.94 had higher resistance to sodium hypochlorite than control cells.

When *Listeria monocytogenes* experienced food-related stress conditions such as NaCl, sodium diacetate, and sodium lactate, its virulence characteristics including invasiveness were increased [12]. Prior exposure of *L*. *monocytogenes* to mild heat stress and tryptone also augmented the bacterial invasion into human epithelial cells [13, 14].

Therefore, the objective of this study was to elucidate the effects of NaCl on heat resistance, antibiotic susceptibility, and Caco-2 cell invasion efficiency of *Salmonella*.

## 2. Materials and Methods

### 2.1. Preparation of *Salmonella* Inoculum

The isolated colonies of *S*. *typhimurium* NCCP10812 and *S*. *enteritidis* NCCP12243 on XLD agar (Difco, Becton Dickinson and Company, Sparks, MD, USA) were cultured in 10 mL tryptic soy broth (TSB; Difco ) at 35°C for 24 h. A fraction (0.1 mL) of the initial culture was then transferred into 10 mL fresh TSB for subculture at 35°C for 24 h, following centrifugation at 1,912 ×g, and 4°C for 15 min. The resulting pellets were washed with phosphate buffered saline (PBS, pH 7.4; 0.2 g of KH_2_PO_4_, 1.5 g of Na_2_HPO_4_, 8.0 g of NaCl, and 0.2 g of KCl in 1 L of distilled water) twice and diluted in PBS to 4–6 Log CFU/mL of inoculum. 

### 2.2. Exposure of *Salmonella* Cells to NaCl

To expose *S*. *typhimurium* NCCP10812 and *S*. *enteritidis* NCCP12243 to NaCl, 0.1 mL portions of the inocula were inoculated into 0, 2 and 4% NaCl (w/v) supplemented with 10 mL TSB and incubated at 35°C for 24 h to obtain stationary phase cells. After exposure to NaCl, 0.1 mL of the cultures were plated on tryptic soy agar (TSA; Difco) containing 0, 2, and 4% NaCl and incubated at 35°C for 24 h to obtain only NaCl-habituated *Salmonella* cells. 

**Figure 1:**
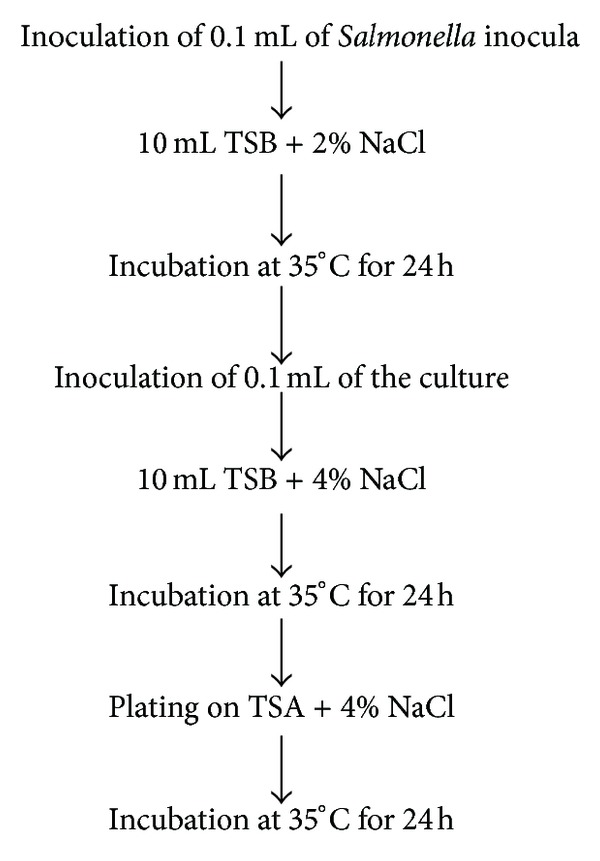
Graphical representation of the preparation of *Salmonella* cells exposed to sequential increase of NaCl concentration.

*Salmonella* cells of inocula were also exposed to a sequential increase of NaCl concentration up to 4% according to the procedure shown in Figure 1. After the incubation of the plates at 35°C for 24 h, 4 mL PBS was added directly on the plates, and the colonies were collected by scraping with a glass rod. The collected bacterial cells were centrifuged (1,912 ×g, and 4°C, for 15 min), and the pellets were washed with PBS twice. These bacterial cell suspensions were then adjusted to OD_600_ = 0.1 with PBS for the heat resistance and antibiotic susceptibility assays, or to OD_600_ = 0.03–0.04 for the Caco-2 cell invasion assay.

### 2.3. Heat Resistance

One milliliter of each bacterial cell suspension was inoculated into 9 mL TSB at 60°C in a water bath. Samples (1 mL) were withdrawn every 20 minutes for 1 h and diluted with 0.1% buffered peptone water (BPW, Difco). The diluents (0.1 mL) were spread-plated on TSA, and the plates were incubated at 35°C for 24 h.

### 2.4. Antibiotic Susceptibility

Antibiotic susceptibility was examined by a disc diffusion assay on Mueller-Hinton agar (MHA, Difco) according to the standard procedure outlined in the National Committee for Clinical Laboratory Standards guidelines [15]. A sterile swab was dampened with the bacterial cell suspensions and then spread on the surface of MHA. The MHA plates were then left at room temperature for 10–15 min, and antibiotic discs (Oxoid, Thermo Fisher Scientific, Basingstoke, Hampshire, UK) were placed on their surface using a multidisc dispenser (Oxoid). The tested antibiotics were amoxicillin (10 *μ*g), chloramphenicol (30 *μ*g), ciprofloxacin (5 *μ*g), gentamicin (10 *μ*g), neomycin (30 *μ*g), oxytetracycline (30 *μ*g), streptomycin (10 *μ*g), and tigecycline (15 *μ*g). After the incubation of the plates at 35°C for 24 h, a portion of clear zone in the order of millimeters around each disc was measured.

### 2.5. Caco-2 Cell Invasion Assay

The bacterial cell suspensions of *S*. *typhimurium* NCCP10812 and *S*. *enteritidis* NCCP12243 were then diluted to 5 × 10^5^–5 × 10^6^ CFU/mL with PBS, and 0.5 mL of the diluents was inoculated into 4.5 mL Eagle's minimum essential medium (MEM medium, Gibco, Penrose, Auckland, New Zealand) supplemented with 20% fetal bovine serum (FBS, Gibco) to be used as inoculum. The inoculum (1 mL) was then inoculated in a monolayer cell of Caco-2 cells (5 × 10^4^ cells/mL) and incubated in 5% CO_2_ at 37°C for 4 h. The upper layer of MEM medium was discarded, and the cells were further incubated in fresh MEM medium supplemented with 20% FBS and 50 *μ*g/mL gentamicin at 37°C for 2 h. After the incubation, the upper layer of the media was discarded, and the Caco-2 cells were washed with PBS twice. A solution (1 mL) of 0.5% Triton X-100 was then added into each well of the plate, and the plate was left on ice for 20 min. The resulting suspension was plated on TSA to enumerate invaded *Salmonella* populations. Invasion efficiency of *Salmonella* to Caco-2 cells was calculated by the equation suggested by Garner et al. [12] as follows:

(1) invasion efficiency (%)=(number  of  Salmonella  cells  invading     Caco-2  cells(CFU/mL)   ×(initial cell counts of   Salmonella(CFU/mL))−1)×100.

### 2.6. Statistical Analysis

The experiment was repeated twice with three samples per repeat (*n* = 6). Bacterial populations were converted to Log CFU/mL before statistical analysis. The data values were analyzed with the general linear model procedures of SAS version 9.2 (SAS Institute Inc., USA). The least squares (LS) means among fixed effects were compared using the “pdiff” option to analyze all the pairwise comparisons at alpha = 0.05 [16].

## 3. Results and Discussion

### 3.1. Heat Resistance

*S*. *typhimurium* NCCP10812 exposed to single NaCl concentrations (0, 2, and 4%) in TSB exhibited increased heat resistance (*P* < 0.05) as the NaCl concentration increased after 60 min of heat challenge. Similarly, Yuan et al. [17] also showed that the adaptation of *S*. *typhimurium* to 5% sodium lactate and 3% sodium acetate enhanced its ability to survive under heat stress. This protective effect of NaCl could be due to a decrease in water activity following NaCl addition. In fact, NaCl causes a poor penetration of heat through the heating medium [18]. However, the heat resistance of *S*. *enteritidis* NCCP12243 was not increased upon increasing concentrations of NaCl (Table 1). Moreover, when the *Salmonella* strains experienced a sequential increase of NaCl concentration, their heat resistances were generally not different from the control (0% NaCl) (Table 1). This result indicates that the effect of NaCl on increased heat resistance of *Salmonella* depends on the specific strain.

### 3.2. Antibiotic Susceptibility

To evaluate the effect of NaCl on antibiotic susceptibility, *S*. *typhimurium* NCCP10812 and *S*. *enteritidis* NCCP12243 were exposed to single concentrations of NaCl or sequentially increased NaCl concentration up to 4%. Exposure of the *Salmonella* strains to single concentrations of NaCl did not change their antibiotic susceptibility (Table 2). However, the antibiotic susceptibility of *S*. *typhimurium* NCCP10812 significantly increased (*P* < 0.05) against chloramphenicol, gentamicin, and oxytetracycline after exposure to sequentially increased NaCl concentration (Table 2). Similarly, *S*. *enteritidis* NCCP12243 also displayed (*P* < 0.05) increased resistance to amoxycillin and ciprofloxacin, while its antibiotic susceptibility against chloramphenicol, gentamicin, oxytetracycline, and streptomycin decreased (*P* < 0.05) after sequential exposure to 4% of NaCl (Table 2). This finding suggests that consistent exposure of *Salmonella* strains to NaCl may alter their antibiotic susceptibility, but the influence of NaCl on the susceptibility of *Salmonella* against antibiotics is dependent on both the strains and antibiotics. Hengge-Aronis [19] found that RpoS, a general stress response regulator, was upregulated when bacteria were exposed to osmotic stress. A study by Huang et al. [20] showed that RpoS has an effect on antibiotic resistances of *Escherichia coli* against ampicillin, chloramphenicol, and rifampicin. Therefore, it can be hypothesized that NaCl may regulate RpoS expression, thereby influencing antibiotic senstivity of *Salmonella* NCCP10812 and NCCP12243.

### 3.3. Caco-2 Cell Invasion Assay

**Figure 2:**
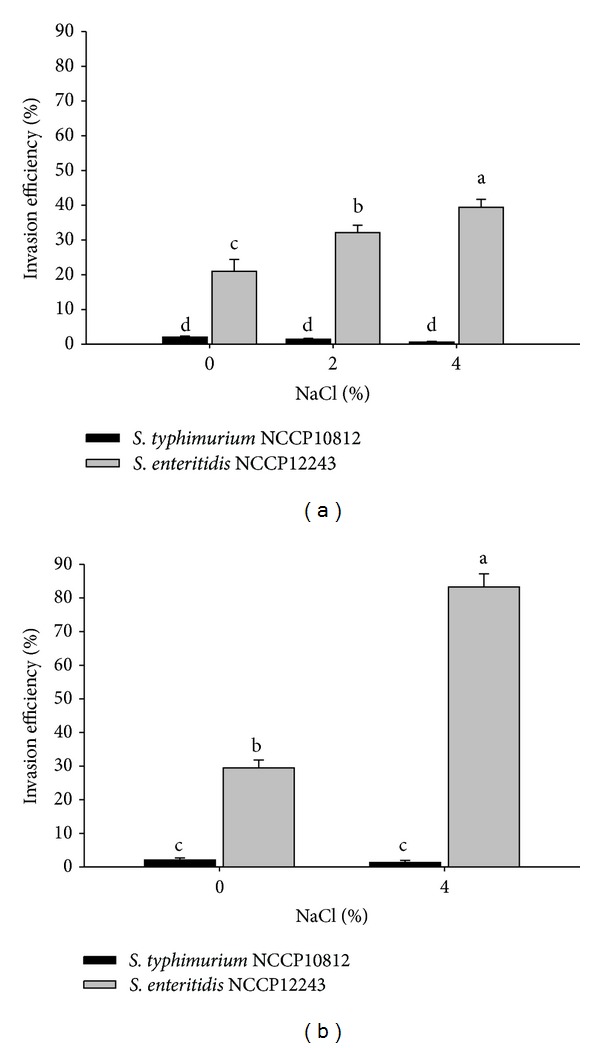
Invasion efficiency of *S. typhimurium* NCCP10812 and *S. enteritidis* NCCP12243 exposed to single concentrations (0, 2, and 4%) of NaCl (a) and sequentially increased NaCl concentrations up to 4% (b). ^a–d^Means with different superscript letters are different (*P* < 0.05).

The cell invasion efficiency of *S*. *typhimurium* NCCP10812 was not affected by NaCl, while *S*. *enteritidis* NCCP12243 showed increased (*P* < 0.05) invasion efficiency into Caco-2 cells from 21% to 39.4% (Figure 2(a)). When *S*. *enteritidis* NCCP12243 experienced a sequential increase of NaCl concentration up to 4%, the invasion efficiency dramatically increased (*P* < 0.05) from 29.5% to 83.3% (Figure 2(b)). This result indicates that the prior exposure to NaCl increases Caco-2 cell invasion efficiency of *Salmonella*, especially when the pathogen is previously exposed to a sequential increase of NaCl concentration. Similarly, *Salmonella* was also more invasive on Henle 407 cells when grown in high osmolarity (0.3 M NaCl) than in low osmolarity (0.06 M NaCl) [21]. Moreover, a study by Sirsat et al. [22] showed that stress such as heat increased *Salmonella* adhesion on Caco-2 cells from 6.3 to 11.7%.

In conclusion, the effect of NaCl on the heat resistance of *Salmonella* is strain dependent, and the sequential increase of NaCl concentration may influence the antibiotic susceptibility of the bacteria. Additionally, NaCl may increase the invasion efficiency of *Salmonella* strains used in this study into Caco-2 cells.

## Figures and Tables

**Table 1:** Reductions (mean ± SD; log⁡(*N*_0_/*N*_*t*_)) of *S. typhimurium* NCCP10812 and *S. enteritidis* NCCP12243, which were exposed to single concentrations (0, 2, and 4%) of NaCl and sequentially increased NaCl concentrations up to 4%, during heat challenge at 60°C for 60 min.

*Salmonella* strains	Heating time (minutes)	Single concentration of NaCl (%)			Sequential increase of NaCl (%)	
		0	2	4	0→0	0→4
*S. typhimurium* NCCP10812	0	0.0 ± 0.0^Aa^	0.0 ± 0.0^Aa^	0.0 ± 0.0^Aa^	0.0 ± 0.0^Zz^	0.0 ± 0.0^Zz^
	20	−3.7 ± 0.3^Ba^	−3.8 ± 0.2^Ba^	−3.9 ± 0.3^Ba^	−3.5 ± 0.2^Yz^	−4.4 ± 0.1^XWy^
	40	−4.6 ± 0.2^Ca^	−4.4 ± 0.1^Ca^	−4.4 ± 0.4^Ca^	−4.3 ± 0.2^Xz^	−4.4 ± 0.7^XWz^
	60	−5.3 ± 0.4^Db^	−4.3 ± 0.1^Ca^	−4.4 ± 0.5^Ca^	−4.8 ± 0.2^Wz^	−4.5 ± 0.6^XWz^

*S. enteritidis* NCCP12243	0	0.0 ± 0.0^Aa^	0.0 ± 0.0^Aa^	0.0 ± 0.0^Aa^	0.0 ± 0.0^Zz^	0.0 ± 0.0^Zz^
	20	−3.6 ± 0.1^Ba^	−3.7 ± 0.4^Ba^	−4.0 ± 0.0^Ba^	−3.5 ± 0.0^Yz^	−3.9 ± 0.4^Yz^
	40	−4.5 ± 0.3^CDb^	−4.6 ± 0.3^Da^	−4.7 ± 0.1^Ca^	−4.0 ± 0.4^Xz^	−4.3 ± 0.2^Xz^
	60	−5.0 ± 0.6^Ca^	−4.9 ± 0.5^CDa^	−4.6 ± 0.2^Ca^	−5.1 ± 0.2^Wz^	−4.8 ± 0.3^Wz^

^A–E^Means within the same column with different superscript letters are different (*P* < 0.05). ^a-b^Means within the same row with different superscript letters are different (*P* < 0.05). ^W–Z^Means within the same column with different superscript letters are different (*P* < 0.05). ^y–z^Means within the same row with different superscript letters are different (*P* < 0.05).

**Table 2:** Diameters (mean ± SD; mm) of clear zones formed by *S. typhimurium* NCCP10812 and *S. enteritidis* NCCP12243 to single concentrations (0, 2, and 4%) of NaCl and sequentially increased NaCl concentrations up to 4%.

*Salmonella* strains	Antibiotic	Single concentration of NaCl (%)			Sequential increase of NaCl (%)	
		0	2	4	0→0	0→4
*S. typhimurium* NCCP10812	Amoxycillin	28.0 ± 0.8	27.5 ± 0.6	27.3 ± 0.5	29.0 ± 0.0^A^	29.5 ± 0.6^A^
	Chloramphenicol	31.8 ± 1.0	33.3 ± 1.5	34.0 ± 1.2	33.3 ± 1.0^A^	12.0 ± 2.4^B^
	Ciprofloxacin	47.0 ± 1.2	49.5 ± 3.4	48.0 ± 2.3	48.0 ± 1.6^A^	46.5 ± 1.9^A^
	Gentamicin	26.3 ± 1.0	26.8 ± 1.0	26.8 ± 1.0	26.5 ± 0.6^A^	14.3 ± 2.2^B^
	Neomycin	22.0 ± 0.8	22.0 ± 0.8	22.3 ± 0.5	24.0 ± 2.2^A^	22.3 ± 2.1^A^
	Oxytetracycline	26.8 ± 4.9	27.0 ± 4.7	27.0 ± 3.6	30.5 ± 1.7^A^	9.5 ± 1.9^B^
	Streptomycin	19.0 ± 0.0	19.3 ± 0.5	19.3 ± 1.0	20.3 ± 1.5^A^	19.5 ± 1.3^A^
	Tigecycline	25.5 ± 1.0	25.8 ± 1.0	26.3 ± 0.5	25.8 ± 0.5^A^	26.8 ± 1.5^A^

*S. enteritidis* NCCP12243	Amoxycillin	27.8 ± 0.5	28.0 ± 0.0	26.8 ± 2.2	28.8 ± 1.3^A^	21.3 ± 8.4^B^
	Chloramphenicol	10.8 ± 2.2	9.8 ± 0.5	11.0 ± 1.6	10.3 ± 2.1^B^	30.8 ± 2.8^A^
	Ciprofloxacin	44.5 ± 6.4	44.0 ± 4.6	39.3 ± 2.2	43.5 ± 4.1^A^	38.0 ± 6.9^B^
	Gentamicin	13.3 ± 0.5	13.5 ± 1.3	12.5 ± 0.6	13.8 ± 1.7^B^	23.8 ± 5.6^A^
	Neomycin	20.5 ± 0.6	21.0 ± 0.0	21.0 ± 0.8	21.0 ± 0.8^A^	24.3 ± 2.4^A^
	Oxytetracycline	8.0 ± 0.0	8.0 ± 0.0	8.0 ± 0.0	8.0 ± 0.0^B^	29.8 ± 1.3^A^
	Streptomycin	16.8 ± 0.5	15.0 ± 2.0	15.0 ± 0.8	16.0 ± 0.8^B^	25.5 ± 4.8^A^
	Tigecycline	25.0 ± 1.2	25.3 ± 1.0	26.8 ± 1.0	26.3 ± 1.0^A^	26.8 ± 1.5^A^

^A-B^Means within the same row with different superscript letters are different (*P* < 0.05).
